# When Do Morally Motivated Innovators Elicit Inspiration Instead of Irritation?

**DOI:** 10.3389/fpsyg.2017.02362

**Published:** 2018-01-12

**Authors:** Jan Willem Bolderdijk, Claire Brouwer, Gert Cornelissen

**Affiliations:** ^1^Department of Marketing, University of Groningen, Groningen, Netherlands; ^2^Department of Economics and Business, Pompeu Fabra University, Barcelona, Spain

**Keywords:** social influence, conformity, morality, ethical consumer choices, innovators, early adopters, social contagion, innovator

## Abstract

Innovators (i.e., consumers who are the first to adopt an innovation) are pivotal for the societal diffusion of sustainable innovations. But when are innovators most influential? Recent work suggests that morally motivated innovators (i.e., consumers who adopt an innovation out of concern for the welfare of others) can make fellow consumers who have not yet adopted that innovation feel morally inadequate. As a self-defense mechanism, those fellow consumers might dismiss these innovators and their choices. As a result, ironically, morally motivated innovators might discourage others to adopt sustainable innovations. In an experimental study, we replicate this pattern, but also show that moral innovators can elicit a more positive response as well. Specifically, our results offer initial evidence that morally motivated innovators may be more inspiring than self-interested innovators, provided that their actions do not directly pose a threat to the moral self-concept of observers. In sum, our research sheds empirical light on the conditions under which innovators are likely to facilitate, rather than slow down the transition to a more sustainable society.

## Introduction

Imagine a man arriving home with a brand new electric car. He stops by his neighbors’ house to show his latest purchase. Sure, the man argues, the car was a bit more expensive, but it does make a valuable contribution to reduce the pollution problem. How would the neighbor respond to the man’s morally motivated purchase decision? Would he be inspired to do the same, or rather discouraged to buy an electric vehicle himself?

### Irritation vs. Inspiration

Innovators – consumers who are among the first to deviate from the status quo (Rogers, 2003), by purchasing for instance an electric car, boycotting brands that use environmentally harmful packaging, or installing solar panels on their roof – play a pivotal role in the transition to a more sustainable society. Their public behavior has the potential to speed up the diffusion of sustainable innovations. First, innovators provide visibility for novel sustainable product alternatives, and thus increase the likelihood that others notice and consider adopting these alternatives (Starr, 2009; Bollinger and Gillingham, 2012). Moreover, they provide social proof – their example can pave the road to adoption for risk averse fellow consumers. Finally, they can impact others unconsciously: consumers are inclined to conform to the perceived norm (Goldstein et al., 2008), and mimic others’ choices (Griskevicius et al., 2012). In short, innovators can inspire fellow consumers to act sustainably as well.

Innovators may adopt a specific sustainable innovation out of collective (‘moral’) or individualistic (‘self-interested’) concerns. People who contribute to the collective good are often applauded by observers (Gouldner, 1960; Hardy and Van Vugt, 2006; Griskevicius et al., 2010), especially when they make their contribution (e.g., buying an electric vehicle) based on moral (e.g., reduced emissions), instead of self-interested concerns (e.g., a tax exemption; Lin-Healy and Small, 2012). Given that observers are more likely to imitate innovators that are considered likeable (Byrne, 1969; Jiang et al., 2010), moral innovators, more so than self-interested innovators, should be influential and inspire observers to follow their example. Additionally, research showed that witnessing another person’s selfless behavior is known to cause a sense of *elevation* (Haidt, 2003; Algoe and Haidt, 2009), an emotion that inspires observers, and mobilizes them to emulate that virtuous behavior.

In sum, these findings suggest that innovators may encourage fellow consumers to make a similar sustainable choice, particularly when the innovator’s deviant choices are motivated by moral concerns.

However, admiration, and feeling inspired, is not the only conceivable response to morally motivated innovators. Recent studies suggest that morally motivated innovators can, ironically, discourage fellow consumers from following their example (Zane et al., 2016). Why would that be the case? The moral self is a central component of people’s overall self-concept. People like to think of themselves as ethical, fair and righteous (Aquino and Reed, 2002). However, consumers’ moral preferences do not always translate into action (e.g., Auger and Devinney, 2007). People’s moral imperfections become painfully salient when they are confronted with fellow consumers who, out of moral concerns, did adopt a sustainable innovation that they failed to adopt themselves. In other words, morally motivated innovators can produce discomfort because they implicitly threaten the moral self-concept of observers (Monin and Jordan, 2009; Cramwinckel et al., 2013). One common way to neutralize this threat is by engaging in defensive processing (Täuber et al., 2014), which may manifest itself in derogating the source of the threat (Fein and Spencer, 1997): the innovator. In other words, observers may dismiss morally motivated innovators, and their choices, in an act of self-preservation (Monin et al., 2008).

Thus, rather than being admired for their efforts to further the collective good, morally motivated innovators can be perceived as threatening, and become a target for denigration and ridicule. Innovators are particularly likely to elicit defensive responses when their atypical choices challenge observers’ relative moral standing. Indeed, meat-eating participants were more likely to derogate a vegetarian fellow consumer who refused to eat meat out of moral (“animal welfare”) instead of self-interested (“dislike the taste”) motives (Cramwinckel et al., 2013).

The findings of Zane et al. (2016) suggest that “do-gooder derogation” can slow down the diffusion of sustainable innovations. Participants who preferred to remain ‘willfully ignorant’ regarding the working conditions under which apparel was produced tended to denigrate others who, unlike them, did choose to seek out and consider such ethical information in their purchase decision. They dismissed these more morally motivated consumers as being ‘boring’ and not ‘sexy.’ Importantly, this act of denigration was found to have important downstream consequences: as people derive their attitudes from their actions (Bem, 1972), ‘denigrators’ subsequently concluded that they apparently “do not care too much about ethical standards” (Zane et al., 2016). As a result, they showed reduced anger toward unethical companies, and, importantly, were less inclined to support ethical consumer alternatives.

### Self-Involvement

In sum, previous literature suggests that morally motivated innovators can elicit either irritation or inspiration among observers. This means that they either act as facilitators of, or impediments to, the diffusion of novel ethical consumption alternatives. However, the conditions under which innovators produce either effect are not well understood. Based on our reasoning above, we argue that morally motivated innovators are less likely to elicit irritation when their behavior does not directly threaten the moral self-concept of observers. When is that the case?

In the studies by Zane et al. (2016), observers were ‘self-involved’ (Cramwinckel et al., 2015): willfully ignorant participants were exposed to others who, in the exact same situation, *did* choose to seek out ethical product information. In that case, drawing a negative social comparison is inevitable for observers: the ethical choices made by others are directly diagnostic for the self-evaluations of observers. When observers are ‘self-involved’ (when their moral self-concept is on the line), derogation is a functional response, as it helps to neutralize the resulting threat. The setting in those studies may correspond to naturally occurring circumstances, as for example in the case of a consumer who decides against the purchase of a fair trade alternative, and subsequently witnesses a fellow consumer who *does* buy the fair trade product.

In many other situations, however, the choice of a morally motivated innovator does not necessarily have much bearing on an observer’s self-concept. That is the case, for example, when the observer has not yet actively considered the ethical alternative in question before witnessing the innovator’s choice. Product innovations form a specific instance of that scenario. If a sustainable innovation is completely novel to observers, and they never had the chance to consider adopting it, observing someone else’s decision to adopt that innovation does not produce a negative social comparison on the side of the observer, nor a self-concept threat. This is a situation where participants lack self-involvement. Because observers do not feel threatened in these situations, there is no reason for them to engage in defensive processing, and no motive to derogate or dismiss the innovator.

We argue that in such neutral situations, innovators are more likely to be inspired by observing the behavior of innovators. Liberated from the need to engage in self-protective reasoning, observers can now evaluate the innovator’s merit from a psychologically safe position. From that position, especially morally motivated innovators might be inspiring to observers, as morally motivated people are typically perceived as more benevolent and admirable than those who are perceived to act in their own self-interest (cf. Lin-Healy and Small, 2013). Because individuals are more likely to imitate whom they like (Byrne, 1969; Jiang et al., 2010), they might be especially likely to imitate the behavior of morally motivated innovators (cf. Lin-Healy and Small, 2013).

In conclusion, we predict that morally motivated actions can provoke admiration and imitation (Haidt, 2003; Algoe and Haidt, 2009) when observers are not self-involved. In that case, we expect that morally motivated innovators will not elicit irritation, but rather inspiration.

### The Current Research

Recent studies (Zane et al., 2016) documented that morally motivated innovators can discourage fellow consumers from choosing the sustainable alternative in situations where observers are self-involved; they experience the innovator’s action as a threat to their moral self-concept. We build on this work, but focus on the possibility of a more positive outcome: when the innovator’s action does not directly implicate the moral self-concept of observers, innovators will not be perceived as threatening. With this threat out of the way, innovators can potentially inspire fellow consumers, especially when observers attribute the innovator’s actions to moral convictions. Thus, the response to sustainable innovators may be more positive among “neutral observers” – those who stand on the sideline, and witness the innovators’ morally motivated choices from a psychologically ‘safe’ distance.

In summary, we predict that self-involvement moderates the response that morally motivated innovators elicit. When observers are not self-involved, morally motivated innovators will be evaluated more positively than self-interested innovators (Hypothesis 1), and may even trigger inspiration (Hypothesis 2). We predict the opposite pattern for self-involved observers: morally motivated innovators are evaluated less positively than self-interested innovators, and demotivate observers to imitate them.

We tested these predictions in the context of a new, ethical consumer innovation – the no-packaging grocery store. No-packaging grocery stores require customers to bring their own containers to transport their purchases. Because products are not pre-packaged, containers can be re-used, and consumers can purchase exactly the amount they need, waste production is reduced compared to traditional retail options. This concept is relevant for our study in that consumers can justify their decision to endorse this store with either morally motivated (i.e., environmental benefits) or self-interested (i.e., monetary benefits) reasons. Moreover, it captures a situation that is fundamentally different from the situations examined previously: unlike familiar ethically-conscious products (Zane et al., 2016), the no-packaging store is a new concept that consumers most likely have not considered themselves. Thus, consumers are in the comfortable position of observing the choice of an innovator, free of threat to their self-concept.

## Materials and Methods

The study employed a 2 (innovator motivation type: moral vs. self-interested) × 2 (observer: self-involved vs. neutral) between subjects design. We measured participants’ evaluation of the innovator, level of self-threat, and liking of the no-packaging grocery store concept. Using Qualtrics, we created a link to an online survey (both in Dutch and English), which was posted on the second author’s Facebook page. The survey was distributed via Facebook to make sure that only experienced Facebook users, who are used to similar invitations, would participate in the study. Participants were blind to the hypotheses. Data were collected between May 9th and June 7th, 2015. Using a snowball procedure, 284 participants started the survey (266 chose the Dutch version, 18 chose the English version). The Ethical Committee of the Faculty of Business and Economics (University of Groningen) provided approval for this study. Written informed consent was obtained from all participants.

### Procedure

In a first phase, all participants were informed about the existence and the characteristics of the “no-packaging grocery stores,” including an illustration.

**FIGURE 1:**
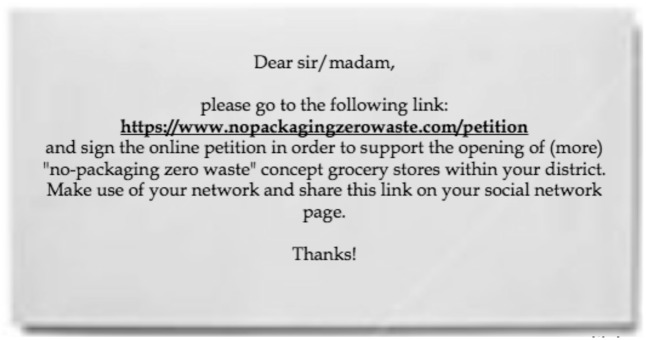
Request to support the “no-packaging” store.

Then, we manipulated self-involvement: half of the participants were asked to consider whether they would comply with a request to endorse the opening of more of these no-packaging grocery stores (*self-involved observer condition*; see **Figure 1**). Complying with the request was deliberately presented as effortful: it involved following a link to a website where they would be required to input their personal information, sign an online petition, and share the link to that petition on their social network profile. As intended, the large majority of participants in the self-involved condition (84%) indicated that they would *not* comply with such a request. The other half – those in the *neutral condition* – did not see this request prior to being exposed to the innovator. As a result, this manipulation created a group of participants who had the opportunity to endorse an ethical innovation, but chose against it (i.e., the self-involved observer condition), and a group of participants who never had the chance to consider endorsing the innovation (i.e., the neutral observer condition).

**FIGURE 2:**
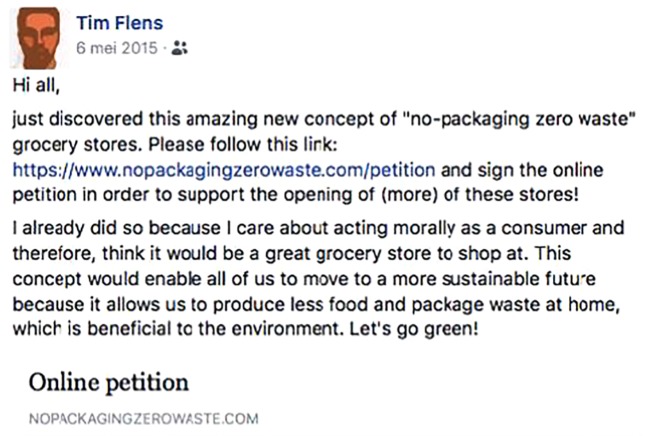
Morally motivated comment.

**FIGURE 3:**
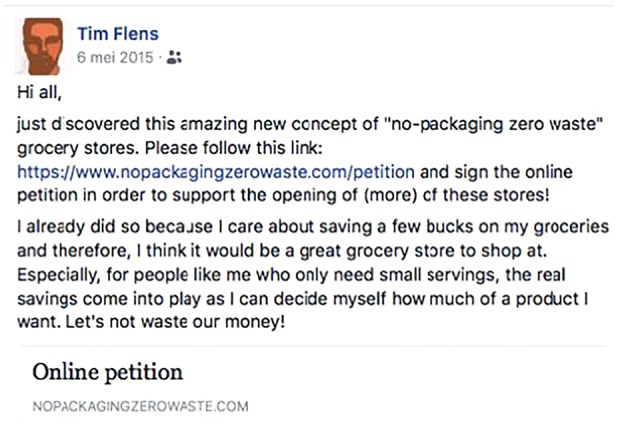
Self-interested comment.

Next, we introduced a fictitious innovator to participants, named ‘Tim,’ via a Facebook post, see **Figures 2**, **3**. Participants read the Facebook post of Tim, who introduced himself as a fellow student, and who had decided to sign the petition endorsing the no-packaging grocery stores. In his post, he invites others to sign the petition as well. We systematically varied the content of Tim’s Facebook post. In the *morally motivated* condition, Tim is introduced as “having a great affinity with sustainability and strives to act in accordance with what is best for the environment,” and in his post, he explains that he decided to support no-packaging grocery stores because of environmental and moral reasons (**Figure 2**). His post ends with the slogan “let’s go green!”. In the *self-interested* condition, Tim is introduced as someone who “keeps track of his expenses as he has a monthly budget that he needs to respect.” In his post, Tim explains that he signed the petition because this new retail model allows him to save money by buying the quantities he needs (**Figure 3**). His post ends with the slogan “let’s not waste our money!”.

In the *self-involved observer condition*, we further emphasized the similarities between Tim and the participant by explaining that Tim “also received the flyer and therefore also had the opportunity to sign the online petition. In contrast to you, Tim did choose to sign the online petition and share the link on his Facebook wall.” In the *neutral* condition, instructions merely introduced Tim as someone who had “signed an online petition to endorse the opening of (more) no-packaging zero waste grocery stores and shared the link on his Facebook wall.”

After being introduced to Tim, participants answered a series of questions that, respectively, measured (1) participants’ evaluations of Tim, (2) participants’ perceived level of self-threat and, after completing a manipulation check, (3) participants’ liking of the no-packaging grocery store concept. The latter was a proxy for inspiration, as it allowed us to evaluate whether being exposed to Tim would increase or decrease participants’ intention to shop in no-packaging grocery stores.

### Evaluations of the Innovator

To measure participants’ evaluations of the Tim, we asked them to complete fourteen 7-point bipolar items (Monin et al., 2008), evaluating Tim in terms of: stupid–intelligent, weak–strong, insecure–confident, passive–active, cruel–kind, awful–nice, cold–warm, dishonest–honest, unfair–fair, unpleasant–pleasant, dependent–independent, stingy–generous, immature–mature, and low self-esteem–high self-esteem. For a further assessment of Tim, participants were asked to indicate on a 7-point Likert scale [from 1 (strongly disagree) to 7 (strongly agree) to what extent they would (a) like Tim as a friend, (b) like Tim as a colleague, and (c) respect Tim as a person]. The combined evaluations yielded a reliable scale (α = 0.97, *M* = 5.05, *SD* = 1.10).

### Perceived Self-Concept Threat

Given that direct questions may give rise to social desirable reponse patterns, we measured self-concept threat indirectly by examing participants’ self-regard (cf. Cramwinckel et al., 2013). Specifically, we asked participants to report to what extent they felt happy with themselves, satisfied with themselves, good, happy, comfortable, confident, determined, disappointed with themselves (reverse-coded), annoyed with themselves (reverse-coded), disgusted with themselves (reverse-coded), angry with themselves (reverse-coded), dissatisfied with themselves (reverse-coded), self-critical (reverse-coded), and guilty (reverse-coded), on a 7-point Likert scale with 1 (totally not applicable) to 7 (totally applicable). Answers to these questions were averaged to form one scale (α = 0.93, *M* = 5.50, *SD* = 1.06).

### Liking of the No-Packaging Store

We finally gauged observers’ liking of the “no-packaging” grocery stores, as a means to examine Tim’s potential to inspire others. We asked participants about the likelihood of them making a purchase at the “no-packaging” store (1 – Extremely unlikely to 7 – Extremely likely) and to which extent this retail concept appealed to them (1 – Not at all to 7 – Extremely). These items formed a reliable index (α = 0.82, *M* = 4.87,*SD* = 1.42).

### Manipulation Check

We included a manipulation check, to ascertain whether participants had read the stimulus material carefully. Participants were asked to check the box that described why Tim supported the concept store. Answer options were (a) it saves money, (b) it is better for the environment, and (c) it offers his favorite products.

## Results

From the 284 participants that started the survey, 22 did not finish, and 29 failed the manipulation check. Given that we are particularly interested in the responses of participants who made a *different* choice than our innovator, we additionally excluded 19 participants who agreed to sign the petition in favor of the no-packaging store^1^. Hence, 214 participants were included in the analysis. Given the effect sizes reported in previous peer evaluation studies (Monin et al., 2008), our sample size is expected to yield a power between 0.88 and 0.99 in detecting the hypothesized effects (Maxwell et al., 2018). Most participants (57.5%) were between the ages of 21 and 30 years old, and 53.3% were female.

We first examined the main and interaction effects of motivation type and self-involvement on innovator evaluation, participants’ self-regard, and store liking, and subsequently examined the underlying process via mediation analysis.

### Innovator Evaluation

**FIGURE 4:**
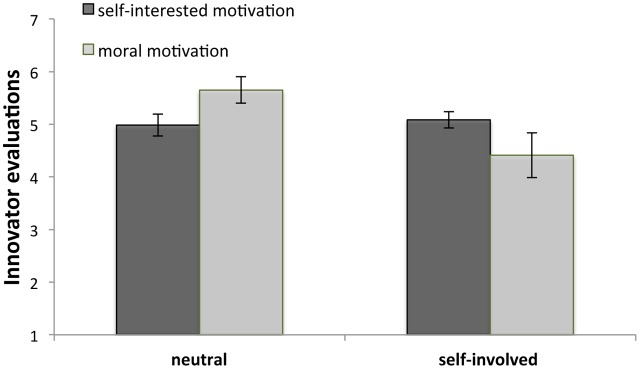
Moral innovators were preferred to self-interested innovators by neutral onlookers, but not by self-involved onlookers. Error bars denote 95% confidence intervals.

We performed a 2 (motivation type: morally motivated vs. self-interested innovator) × 2 (self-involved vs. neutral observers) ANOVA with innovator evaluation as the dependent variable. There was no main effect of motivation type, *F*(1,210) = 0.000, *p* = 0.98, but we found a main effect of self-involvement, *F*(1,210) = 16.98, *p* < 0.001. Participants in the neutral condition (*M* = 5.33, *SD* = 0.93) evaluated Tim more positively than those in the self-involved observer condition (*M* = 4.74, *SD* = 1.19). Importantly, we found the expected interaction between motivation type and self-involvement, *F*(1,210) = 23.45, *p* < 0.001, see also **Figure 4**. *Post hoc* comparisons (using Tukey’s HSD test) revealed that those in the self-involved observer condition – those who could have but did not sign the petition – evaluated Tim more negatively when he employed morally motivated (*M* = 4.41, *SD* = 1.52) instead of self-interested arguments (*M* = 5.08, *SD* = 0.54), *M*_difference_ = -0.67, *p* = 0.005, Cohen’s *d* = 0.59. We find the opposite pattern among participants in the neutral condition (those who were never offered to sign the petition). They evaluated Tim most positively when he employed morally motivated (*M* = 5.65, *SD* = 0.97) instead of self-interested arguments (*M* = 4.98, *SD* = 0.75), *M*_difference_ = 0.67, *p* = 0.003, Cohen’s *d* = 0.76^2^.

### Self-Regard

**FIGURE 5:**
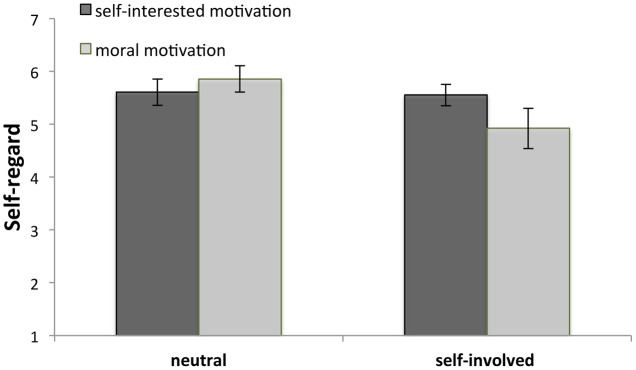
Involved participants felt least satisfied with themselves when they were confronted with a morally motivated innovator. Error bars denote 95% confidence intervals.

We repeated this analysis, now with participants’ self-regard as the dependent variable. There was no main effect of motivation type, *F*(1,210) = 1.86, *p* = 0.17, but there was a main effect of self-involvement, *F*(1,210) = 12.78, *p* < 0.001. Participants in the self-involved condition (*M* = 5.23, *SD* = 1.13) were less satisfied with themselves than participants in the neutral condition (*M* = 5.74, *SD* = 0.95). Importantly, we found the predicted interaction between motivation and self-involvement, *F*(1,210) = 10.08, *p* = 0.002, see **Figure 5**. *Post hoc* comparisons (using Tukey’s HSD test) revealed that participants in the self-involved condition – those who could but did not sign the petition – felt less satisfied with themselves when confronted with the morally motivated (*M* = 4.92, *SD* = 1.36) instead of the self-interested Tim (*M* = 5.55, *SD* = 0.71), *M*_difference_ = -0.63, *p* = 0.01, Cohen’s *d* = 0.58. For participants in the neutral condition, however, there is no effect of motivation type on self-regard – participants are satisfied with themselves, regardless of whether they were confronted with the morally motivated (*M* = 5.86, *SD* = 0.97) or self-interested Tim (*M* = 5.61, *SD* = 0.91), *M*_difference_ = 0.25, *p* = 0.55, Cohen’s *d* = 0.27.

### Liking of the No-Packaging Store

**FIGURE 6:**
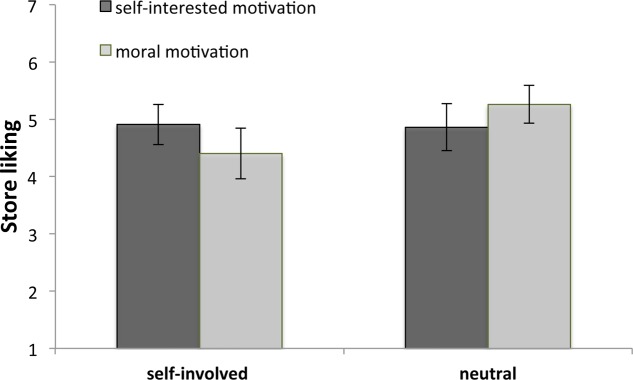
Involved participants were more enthusiastic about the no-packaging store when it was promoted by a self-interested innovator. Neutral participants were more enthusiastic when the store was advocated by a moral innovator. Error bars denote 95% confidence intervals.

Finally, as a way to gauge Tim’s potential to inspire, we examined the effects of self-involvement and motivation type on participants’ liking of the no-packaging store concept. Motivation type did not have an influence on participants’ liking of the store, *F*(1,210) = 0.08, *p* = 0.78, but we again found a main effect of self-involvement, *F*(1,210) = 4.48, *p* = 0.04. Those in the neutral condition (*M* = 5.07, *SD* = 1.39) were more supportive of the novel store concept than those in the self-involved condition (*M* = 4.65, *SD* = 1.43). Importantly, we found the expected interaction effect between motivation type and self-involvement, *F*(1,210) = 5.62, *p* = 0.019, see **Figure 6**. Participants in the self-involved condition evaluated the novel store concept less positively when that store was advocated by the morally motivated Tim (*M* = 4.40, *SD* = 1.57) instead of self-interested Tim (*M* = 4.91, *SD* = 1.24). However, this difference was not statistically significant in a Tukey’s HSD *post hoc* test, *M*_difference_ = -0.51, *p* = 0.27, Cohen’s *d* = 0.36. Participants in the neutral condition were not thrown off by the presence of a morally motivated innovator. If anything, there is a trend that they liked the novel store concept better when it was advocated by the morally motivated (*M* = 5.26, *SD* = 1.27) instead of the self-interested (*M* = 4.86, *SD* = 1.50) Tim, but this difference was not statistically significant, *M*_difference_ = 0.40, *p* = 0.43, Cohen’s *d* = 0.29.

### Mediation Analyses

The results thus far are consistent with our argumentation: participants in the neutral condition – those who are in the comfortable position to observe the innovator’s actions from a psychologically safe distance – are not implicated by the innovator’s choices, tend to evaluate moral innovators positively, and may even develop an interest to imitate their choices. For self-involved observers – those who have considered the ethical alternative, but decided against it – however, derogating morally motivated innovators and their choices is a functional response. When evaluating the innovator, they do not only factor in the benevolence of the innovator, but may also have a need to protect their threatened self-concept.

We examined this process more directly. Specifically, our reasoning implies that self-threat would mediate the effect of motivation type on (1) innovator evaluation and (2) store liking. This process is only relevant for participants in the self-involved condition, as the self-concept of neutral participants is not challenged by morally motivated innovators. Thus, we tested for a conditional mediation effect (Preacher et al., 2007).

Employing model 7 (bias-corrected, 1000 bootstrap samples) of the PROCESS macro (Hayes, 2013), we indeed find that the indirect effect of motivation type via feelings of self-threat on innovator evaluation was significant for participants in the self-involved condition, *b* = -0.38, *SE* = 0.16, 95% CI [-0.74, -0.12], but not for those in the neutral condition, *b* = 0.15, *SE* = 0.11, 95% CI [-0.05, 0.40]. Repeating the analysis with store liking as the dependent variable, we find the same pattern; the indirect effect of motivation type via feelings of self-threat on store liking was significant for those in the self-involved condition, *b* = -0.20, *SE* = 0.16, 95% CI [-0.42, -0.06], but not for those in the neutral condition, *b* = 0.08, *SE* = 0.16, 95% CI [-0.02, 0.24]. Hence, self-threat only takes up a mediating role when participants are self-involved.

These results are consistent with our reasoning: the observed drop in innovator and store liking in the self-involved condition stems from the self-threat that those participants experience when confronted with a morally motivated innovator. In the neutral condition, observers are not bothered by morally motivated innovators because they pose no threat to their own self-concept. Thus, they are in a position to applaud and even be inspired by morally motivated innovators.

## Discussion

Zane et al. (2016) found that morally motivated innovators can elicit irritation and denigration, and discourage other consumers from imitating their sustainable choices. We replicated this phenomenon, and documented a boundary condition. We found support for our reasoning that “do-gooder derogation,” and its consequences, only occurs when innovators’ sustainable choices form a threat to observers’ moral self-concept. When there is no such threat, there is no need for observers to engage in self-preservation, and they evaluate innovators based on the moral merit of their choices.

In line with this reasoning, we found that for self-involved participants, innovators are more likely to be experienced as a threat, to elicit derogation, and to discourage imitation when they justify their choices with morally charged arguments instead of self-interested arguments. On the other hand, when the choices of the innovator do not directly implicate observers, we found that participants evaluated moral innovators more positively than self-interested innovators. This implies that moral innovators are not necessarily impediments to change, but could even act as effective change agents, as long as their choices do not form a threat to the moral self-concept of observers. In other words, morally motivated innovators could potentially inspire others to follow their example. However, the evidence in the current data for such inspirational effect is limited to a non-significant trend. Future research is required to further examine this possibility.

### Process

As suggested in Zane et al. (2016), but not directly tested, our moderated mediation analysis suggests that people denigrate moral innovators in an attempt to restore their threatened self-concept. However, it remains unclear which process is exactly responsible for producing this self-concept threat. The nature of our manipulation allows us to speculate. We included the fictitious Facebook user ‘Tim’ in our experiments, rather than introducing a confederate who was physically present and with whom participants were required to interact (Monin et al., 2008; Cramwinckel et al., 2013). This more subtle exposure to a morally motivated innovator proved sufficient to elicit moral self-concept threat (as evidenced by the significant drop in self-regard among self-involved participants who were exposed to the moral Tim). The fact that such a subtle cue has a measurable impact on participants’ self-regard is telling. Specifically, it suggests that innovators are not disliked because they represent a different set of values, because observers fear that innovators may disapprove of their choices (Minson and Monin, 2012) or because innovators raise the bar for what is appropriate behavior in a certain setting for others (Parks and Stone, 2010). Instead, in line with more recent work (O’Connor and Monin, 2016), our results suggests that moral innovators produce discomfort because of an *internal* process on the side of the observer. Morally motivated innovators activate observers’ pre-existing sense of moral imperfection. Thus, the threat seems to emanate from the fact that innovators can remind fellow consumers of their own moral shortcomings. That interpretation would also explain why especially those who care strongly about morality tend to take offense at moral do-gooders (Cramwinckel et al., 2013). Future research is needed to uncover the exact nature of this mechanism.

### Limitations and Future Research

We did not systematically vary the gender of our male innovator (“Tim”), which may have suppressed the effects – female participants could have felt even more threatened if they were confronted with the actions of morally motivated “Theresa” instead of “Tim.” Follow-up analyses, however, suggest that female participants felt as threatened by Tim as male participants did.

This paper focused on the psychological impact of innovators on observers, and did not include behavioral measures of irritation and inspiration. We considered offering participants the opportunity to revise their earlier decision regarding whether to sign the petition. We would have predicted that in the self-interested motivation condition, a larger proportion of participants would choose to sign the petition when given another chance, compared to the moral motivation condition. However, we decided against it, as this would introduce a confounding mechanism affecting the decision to sign: deciding to sign the petition on the second occasion would be inconsistent with earlier behavior. As a result, signing the petition when the second occasion arises would effectively diagnose participants’ earlier decision not to sign as “wrong.” Surely, that is something what participants would be motivated to avoid. Instead, we used participants’ attitudes toward the no-packaging stores as a proxy for innovators’ potential to inspire others.

Although we have no reason to believe that these results are specifically biased in the direction of our predictions, we welcome future behavioral studies that build on our initial findings and extend this research into for instance an actual retail setting, for example involving in-store customers who are exposed to a real-life confederate who expresses ethical vs. self-interested motivations.

In our studies, we simulated a situation in which an innovator’s moral choices do not imply a threat to the observer, by using an ethical innovation that most consumers have not yet actively considered as stimulus material – the no-packaging store. Since consumers have never declined to adopt this innovation, the innovator’s behavior does not confront them with a moral imperfection. As a result, observers do not proceed to derogating the innovator. There are other factors that influence whether, or to which extent, morally motivated innovators present a threat to observers. For instance, an innovator who makes morally motivated choices might pose less of a threat to observers, if observers know that the innovators have moral imperfections themselves (Howe and Monin, 2017). For example, being confronted with a vegetarian might produce threat and lead to derogation for meat-eaters (Cramwinckel et al., 2013). However, if that vegetarian would claim to eat meat once a year, as a treat to himself, observers may feel less threatened, as their benchmark for evaluating their moral self-concept is no longer “moral perfection.” Finally, innovators may distinguish between the moral value of their act and the person performing it. By claiming something along the lines of “I am making this choice because it works for me, but other people should be free to make their own decisions,” innovators and their choices should be less threatening to observers (Howe and Monin, 2017). Future research should test these predictions.

Finally, by using the no-packaging store concept, we aimed to create a situation in which for the majority of our participants would not feel self-involved. However, we cannot rule out the possibility that some of our participants did know the store concept already, and decided not to shop there. For those participants, the setting in our study would have been one of high, rather than low self-involvement. The presence of such participants would create noise in our data and might be responsible for not finding larger effects than the ones we found here. Future studies should verify whether participants are indeed unfamiliar with the innovation in question, to ascertain all participants lack a sense of self-involvement.

### Implications

In order to secure a sustainable future, it is crucial to develop a better understanding of how policy makers can sway individual consumers to make more sustainable (such as environmentally conscious and socially responsible) consumption choices. A number of recent studies have zoomed in on the persuasive impact of moralization: positioning pro-environmental behavior as an moral imperative has the potential to convince (at least a subpart) of the population to act more sustainably (Bolderdijk et al., 2013). Moralization can be effective because consumers care deeply about maintaining a moral self-concept (Aquino and Reed, 2002), which can be achieved by complying with appeals that are congruent with one’s (idiosyncratic) moral standards (Kidwell et al., 2013).

A much less studied issue pertains to how moral appeals for sustainable behavior affect those who choose *not* to comply. Recent work Täuber et al. (2015) suggests that positioning sustainable behavior as a morally superior choice can have adverse effects on non-compliers: given that they also strive for a moral self-image, non-compliers may look for ways to rationalize their behavior, for instance by actively questioning the veracity of sustainability claims (Campbell and Kay, 2014). So, while encouraging some consumers to act more sustainably, moralization can also trigger skepticism and rebellion among a subset of consumers, and thus further increase polarization.

Although pertaining to interpersonal dynamics, our work, as well as that by Zane et al. (2016), provides an important empirical hint that highlighting consumers’ moral shortcomings –– which is effectively what moral appeals do in the eyes of those who chose not to comply – indeed bears the risk of further intensifying the polarization between those who do and those who do not see sustainability (e.g., halting climate change) as a goal that merits behavior change.

So, should policy makers abandon moralization as a strategy to promote sustainable consumer choices? We believe not, although caution is required. First, as our findings imply, moral appeals have the potential to elicit inspiration and imitation, more so than self-interested appeals. Second, moralization may have an important function in the transition to a more sustainable society – moral appeals can facilitate positive spillover (sustainable behavior in one domain can foster sustainable behavior in related domains, Evans et al., 2013) and can help to shape the emergence of new norms (Thøgersen and Crompton, 2009). Third, in this study, we found negative effects of moralization on the short term. It is possible that the threat caused by moral innovators provoke dismissal at first, but can motivate individuals to follow their example in the longer term. Future studies should test this possibility.

Insights that help policymakers predict when and why moralization is most likely to elicit inspiration rather than rebellion among consumers, is a crucial addition to this literature. In the context of promoting the uptake of ethical alternatives, for instance, policy makers walk a thin line between inspiring and irritating consumers – positioning sustainable products as a moral imperative (“slavery-free chocolate”, “climate-neutral cars”), may unintentionally threaten consumers who were in a position to adopt, but thus far decided against it. As our studies imply, consumers may not feel threatened, and potentially even inspired when policy makers present these alternatives as *innovations* that consumers, due to their novelty, simply could not have considered before.

## Ethics Statement

This study was carried out in accordance with the recommendations of “The Ethics Committee of the Faculty of Business and Economics of the University of Groningen” with written informed consent from all subjects. All subjects gave written informed consent in accordance with the Declaration of Helsinki. The protocol was approved by the “The Ethics Committee of the Faculty of Business and Economics of the University of Groningen”.

## Author Contributions

All authors were involved in writing the article. The data were collected by CB, using a snowball sampling method. This study was part of her Master thesis, which was conducted under the supervision of the JB and GC, during May and June of 2015. The data were analyzed by JB and CB.

## Conflict of Interest Statement

The authors declare that the research was conducted in the absence of any commercial or financial relationships that could be construed as a potential conflict of interest.

## Footnotes

1 The overall pattern of results are similar when including these 19 in the ensuing analyses. Although it would be theoretically interesting to analyze how participants who agreed to the petition would respond to the different pioneer motivations, the limited size of this sample (*N* = 19) precluded us from making useful inferences.

2 Copying the procedure of Monin et al. (2008), we subjected the different items gauging innovator evaluations to a principled axis factor analysis (Promax rotation). The results (using the scree plot) unequivocally suggest a 1-factor solution: 68% of the variance was captured in the first factor. In other words, it seems that participants who experienced threat evaluated Tim negatively across the board, as manifested in lower ratings on various dimensions. This effect may potentially be a manifestation of motivated reasoning – participants, depending on whether they feel threatened, instantly form a specific overall judgment of Tim, which consequently motivates them to also be consistent in the specific dimensions they rated Tim on.
